# A Regularized MANOVA Test for Semicontinuous High‐Dimensional Data

**DOI:** 10.1002/bimj.70054

**Published:** 2025-04-29

**Authors:** Elena Sabbioni, Claudio Agostinelli, Alessio Farcomeni

**Affiliations:** ^1^ Department of Mathematical Science Politecnico di Torino Torino Italy; ^2^ Department of Mathematics University of Trento Trento Italy; ^3^ Department of Economics and Finance Tor Vergata University of Rome Rome Italy

**Keywords:** penalized methods, permutation tests, zero‐inflation

## Abstract

We propose a MANOVA test for semicontinuous data that is applicable also when the dimension exceeds the sample size. The test statistic is obtained as a likelihood ratio, where the numerator and denominator are computed at the maxima of penalized likelihood functions under each hypothesis. Closed form solutions for the regularized estimators allow us to avoid computational overheads. We derive the null distribution using a permutation scheme. The power and level of the resulting test are evaluated in a simulation study. We illustrate the new methodology with two original data analyses, one regarding microRNA expression in human blastocyst cultures, and another regarding alien plant species invasion in the island of Socotra (Yemen).

## 1 Introduction

A well‐known generalization of ANOVA tests for the case of multivariate data is the MANOVA test (Wilks 1938). The test involves a joint null hypothesis claiming equal mean vectors of two or more multivariate Gaussian distributions, usually under an assumption of equal covariance matrices. While classical tests are devised for low‐dimensional settings, there are several contributions for continuous data when the dimension exceeds the sample size, with recent reviews Harrar and Kong (2022). More in detail, some works involve some form of regularization (Cai and Xia 2014; Chen et al. 2011; Dong et al. 2017), in order to guarantee positive definiteness of the (possibly common) variance covariance matrix; other works involve estimation of the traces of covariance matrices (Chen and Qin 2010; Hu et al. 2017; Srivastava and Kubokawa 2013; Yamada and Himeno 2015).

In this work, we are interested in semicontinuous data, that involve positive continuous measurements plus a certain fraction of exactly zero observations. Semicontinuous data are measured in several important applications like abundance estimation in ecology Farcomeni (2016), omics data Taylor and Pollard (2009), cost‐effective analysis in medical research (Tu and Zhou 1999). Inference involves separately modeling the mass probability at zero, and the positive continuous observations conditionally on a non‐zero measurement (Chai and Bailey 2008; Lachenbruch 2001, 2002). A common assumption is that the positive measurements are log‐normal. Tests have appeared for different formulations of the null hypothesis (Tu and Zhou 1999; Xiao‐Hua and Tu 1999; Zhou and Tu 1999), which inevitably involve both the occurrence probabilities and the conditional moments of the continuous part. Formal MANOVA tests for semicontinuous data have appeared for the case in which the dimension does not exceed the sample size (Farcomeni 2016). Here, we propose a MANOVA test for semicontinuous data, based on regularization, that is applicable also when the dimension exceeds the sample size. This is a quite common occurrence in modern applications, including the areas discussed above. To motivate our contribution, we present two original data examples. One involves testing homogeneity of microRNA expression in human blastocysts, where many zeros are observed due to the very early stage of development, and the dimension is large. The other application involves comparing overall abundance of several invasive species in the island of Socotra (Yemen), where many alien species might not be observed in a few sampling spots. To the best of our knowledge, our MANOVA test for high‐dimensional semicontinous data is the first test of this kind to appear in the literature.

The rest of the paper is as follows. In the next section, we set up the general framework and notation. A penalized version of the maximum likelihood estimator (MLE) for the general model is described in Section 2.1. The null hypothesis and the resulting likelihood‐ratio type test are described in Section 2.2. The resulting test is evaluated through a simulation study in Section 3, where we report on the observed level and power over different scenarios. We then illustrate the new methodology through two original real data examples. The microRNA data are described and analyzed in Section 4.1; while the alien species data in Section 4.2. Some concluding remarks are given in Section 5.

The methods discussed in this paper have been implemented in an R package called semicontMANOVA, whose source and compiled version are available along with this paper on the publisher's website.

## 2 General Framework

Assume we have K groups, where group k has $n_{k}$ observations, where k=1,⋯,K. Let $n:=\sum_{k=1}^{K}n_{k}$ denote the total number of observations. Let $X_{k}$ be an $n_{k}\times p$ matrix that contains $n_{k}$ p‐dimensional observations of the kth group. In our framework, each element $X_{ijk}$ can be either zero, or positive, and we expect a positive proportion of zeros.

Consider $Y_{ijk}:=1\left(X_{ijk}>0\right)$, where 1(·) is the indicator function and let $a:=\left(a_{1},\cdots ,a_{p}\right)\in {\left\{0,1\right\}}^{p}$ be any of the possible configurations of presences ($Y_{ijk}=1)$ and absences ($Y_{ijk}=0$) over p components. We model $Y_{ik}=\left(Y_{i1k},\cdots ,Y_{ipk}\right)$ with a multivariate Bernoulli distribution, that is fully specified by $2^{p}-1$ free parameters of the kind $\pi_{k}\left(a\right):=P\left(Y_{i1k}=a_{1},\cdots ,Y_{ipk}=a_{p}\right)$, assuming $\sum_{a\in {\left\{0,1\right\}}^{p}}\pi_{k}\left(a\right)=1$.

For all k=1,…,K, $i=1,\text{…},n_{k}$, and j=1,…,p, we let ${\tilde{X}}_{ijk}:=\log X_{ijk}$ when $Y_{ijk}=1$, otherwise ${\tilde{X}}_{ijk}$ is not observed. We assume that ${\tilde{X}}_{ik}$ follows a multivariate p‐dimensional Gaussian distribution, with mean $\mu_{k}$ and covariance matrix Σ when all the components are observed. We thus make a homoskedasticity assumption.

Estimating all the parameters of the Bernoulli distribution is reasonable when p is small, while their number is definitely not manageable in situations of high dimensionality of the data, especially when p>n. We reduce the number of parameters by assuming that the joint probability $\pi_{k}$ depends only on the number of positive components that are present in one observation. Formally, we assume

(1) $$\pi_{k}\left(a_{r}\right)=\pi_{k}\left(a_{t}\right)\;\text{if}\;a_{r},a_{t}\in {\left\{0,1\right\}}^{p}\;\text{s.t.}\;\sum_{j=1}^{p}a_{jr}=\sum_{j=1}^{p}a_{jt}.$$

To simplify the notation, we define $\pi_{k}\left(s\right):=\pi_{k}\left(a\right)$ for each a such that $s:=\sum_{j=1}^{p}a_{j}$, where s=0,⋯,p. Assuming (1), the number of parameters describing the presence/absence part of the data decreases from $K(2^{p}-1)$ to Kp. Assumption (1) above can be verified up front through a simple Chi‐square test. We let $\theta :=\left(\pi ,\mu ,\Sigma \right)$ be the set of all unknown parameters in the parametric space $\Theta ={\left(0,1\right)}^{Kp}\times R^{Kp}\times S_{p}$, where $S_{p}$ is the set of all symmetric positive definite p×p matrices. The total number of parameters is ϑ=2Kp+p(p+1)/2.

### 2.1 Likelihood Inference

In this section, we introduce a penalized version of the maximum likelihood estimator (MLE) for all the parameters θ. The penalty is necessary for the case in which p>n, and might be useful otherwise (e.g., an increased bias might correspond to a decrease in variance). We define the set $V\left(Y_{ik}\right):=\left\{j\in \left\{1,\cdots ,p\right\}:Y_{ijk}=1\right\}$, containing the indices of all positive (observed) components of $X_{ik}$. In the following discussion, given a p‐dimensional vector b and a set V⊆{1,⋯,p}, $b_{V}$ is the |V|‐dimensional vector with the components of b that are indexed by the elements of V. Similarly, given a p×p matrix A, $A_{V}$ indicates the |V|×|V| matrix built considering only the rows and the columns of A that have indices belonging to V. Hence, let $n_{ik}:=\sum_{j=1}^{p}Y_{ijk}$ the number of observed components for the ith observation in the kth group. We have

(2) $${\tilde{X}}_{ik}|Y_{ik}∼N_{n_{ik}}\left(\mu_{V(Y_{ik})k},\Sigma_{V(Y_{ik})}\right),$$

i.e., conditionally on the presence‐absence vector $Y_{ik}$, the logarithm of the positive components of $X_{ik}$ follows a multivariate Gaussian distribution, with dimension equal to the number of non‐zero components and parameters obtained as the corresponding subset of the p‐dimensional parameters $\mu_{k}$ and Σ. Combining the information from ${\tilde{X}}_{ik}$ and $Y_{ik}$, the likelihood is given by

$$\begin{array}{cc} L(\theta ) & =\prod_{k=1}^{K}\prod_{i=1}^{n_{k}}\prod_{a}\pi_{k}{\left(a\right)}^{1(V\left(a\right)=V\left(Y_{ik}\right))}\times \prod_{k=1}^{K}\prod_{i=1}^{n_{k}}\left\{{\left(2\pi \right)}^{-\frac{n_{ik}}{2}}{\left|\Sigma_{V(Y_{ik})}\right|}^{-\frac{1}{2}}\right.\\ & \times \left.\exp\left[-\frac{1}{2}{\left({\tilde{X}}_{iV(Y_{ik})k}-\mu_{V(Y_{ik})k}\right)}^{⊤}\Sigma_{V(Y_{ik})}^{-1}\left({\tilde{X}}_{iV(Y_{ik})k}-\mu_{V(Y_{ik})k}\right)\right]\right\}\;. \end{array}$$

and the correspoding log–likelihood is

$$\begin{array}{cc} \ell (\pi ,\mu ,\Sigma )=\; & \sum_{k=1}^{K}\sum_{i=1}^{n_{k}}\sum_{a}1\left(V\left(a\right)=V\left(Y_{ik}\right)\right)\log\left(\pi_{k}\left(a\right)\right)\\ & +\sum_{k=1}^{K}\sum_{i=1}^{n_{k}}\left[\frac{-n_{ik}}{2}\log\left(2\pi \right)+-\frac{1}{2}\log\left(\left|\Sigma_{V(Y_{ik})}\right|\right)\right.\\ & \left.-\frac{1}{2}{\left({\tilde{X}}_{iV(Y_{ik})k}-\mu_{V(Y_{ik})k}\right)}^{⊤}{\left(\Sigma_{V(Y_{ik})}\right)}^{-1}\left({\tilde{X}}_{iV(Y_{ik})k}-\mu_{V(Y_{ik})k}\right)\right]. \end{array}$$

When p>n the usual MLE of $\pi_{k}$, $\mu_{k}$ and Σ is not well defined, since the sample variance and covariance matrix are not positive definite. We regularize the problem through a Ridge‐like penalty. Specifically, the regularized log‐likelihood $\ell^{\lambda }\left(\theta \right)$ can be written as

(3) $$\ell^{\lambda }\left(\pi ,\mu ,\Sigma \right)=\ell \left(\pi ,\mu ,\Sigma \right)-\frac{1}{2}P\left(\lambda ,\Sigma \right),$$

where the penalization term P(λ,Σ) is defined as

(4) $$P\left(\lambda ,\Sigma \right):=\sum_{k=1}^{K}\sum_{i=1}^{n_{k}}\text{tr}\left(\Lambda_{V(Y_{ik})}\Sigma_{V(Y_{ik})}^{-1}\right),$$

with $\lambda ={\left(\lambda_{1},\lambda_{2},\text{…},\lambda_{p}\right)}^{⊤}\in R_{+}^{p}$ and Λ=diag(λ). In the low‐dimensional setting with n>p one can set λ=0. The penalized MLEs of the parameters involved in the regularized likelihood (3) can be obtained in the closed form (see details in Appendix A) and are given by

(5) $$\begin{array}{cc} {\hat{\pi }}_{k}\left(s\right) & =\frac{s!(p-s)!}{p!}\frac{\sum_{i=1}^{n_{k}}1\left(|V(Y_{ik})|=s\right)}{n_{k}}, \end{array}$$

(6) $$\begin{array}{cc} {\hat{\mu }}_{jk} & =\frac{\sum_{i=1}^{n_{k}}{\tilde{X}}_{ijk}Y_{ijk}}{\sum_{i=1}^{n_{k}}Y_{ijk}}, \end{array}$$

(7) $$\begin{array}{cc} {\hat{\Sigma }}^{\lambda } & =\frac{\sum_{k=1}^{K}\sum_{i=1}^{n_{k}}\left[Y_{ik}Y_{ik}^{⊤}\circ \left(\left({\tilde{X}}_{ik}-{\hat{\mu }}_{k}\right){\left({\tilde{X}}_{ik}-{\hat{\mu }}_{k}\right)}^{⊤}+\Lambda \right)\right]}{\sum_{k=1}^{K}\sum_{i=1}^{n_{k}}Y_{ik}Y_{ik}^{⊤}}, \end{array}$$

with j=1,…,p and k=1,…,K. Note that the fraction in the variance and covariance matrix estimator is considered to be entry‐wise and ∘ denotes the Hadamard product. When λ=0 the expression above coincides with the MLE Farcomeni (2016). A general requirement is that $\sum_{k=1}^{K}\sum_{i=1}^{n_{k}}Y_{ij_{1}k}Y_{ij_{2}k}>0$ for each $j_{1}\neq j_{2},\;j_{1},j_{2}=1,\cdots ,p$, that is, each couple of variables is simultaneously present in at least one observation in the sample. In real applications with p>>n there might be couples of variables that do not satisfy the previous condition. Those variables must be removed from the dataset starting from those with the largest number of absent components, until all the others satisfy the condition. This process reduces the dimension, but it has an impact only on the continuous part of the likelihood.

### 2.2 Hypothesis Testing

In this section, we define a MANOVA test in our possibly high‐dimensional semicontinuous setting. The null hypothesis we wish to test, under the framework previously described, can be summarized as

$$H_{0}:\left(\cap_{s=0}^{p}\pi_{1}\left(s\right)=\cdots =\pi_{K}\left(s\right)\right)\cap \left(\cap_{j=1}^{p}\mu_{j1}=\cdots =\mu_{jK}\right),$$

which corresponds to assuming the probability of having s positive components, with s=0,⋯,p, is equal in all the groups, and, given a presence, all expectations are the same. We let $\Theta_{0}\subseteq \Theta$ be the parametric space under the null hypothesis. For the sake of generality we admit the possibility of a penalty parameter $\lambda_{0}={\left(\lambda_{01},\lambda_{02},\text{…},\lambda_{0p}\right)}^{⊤}$ under the null hypothesis that can differ from λ, the penalty parameter under the alternative hypothesis. The log‐likelihood under $H_{0}$ can be written as

$$\begin{array}{ccc} & & \ell_{0}\left(\pi_{0},\mu_{0},\Sigma_{0}\right)\\ & & \;=\;\sum_{k=1}^{K}\sum_{i=1}^{n_{k}}\sum_{a}1\left(V\left(a\right)=V\left(Y_{ik}\right)\right)\log\left(\pi_{0}\left(a\right)\right)\\ & & \;+\sum_{k=1}^{K}\sum_{i=1}^{n_{k}}\left[\frac{-n_{ik}}{2}\log\left(2\pi \right)+-\frac{1}{2}\log\left(\left|\Sigma_{V(Y_{ik})0}\right|\right)\right.\\ & & \;\left.-\frac{1}{2}{\left({\tilde{X}}_{iV(Y_{ik})k}-\mu_{V(Y_{ik})0}\right)}^{⊤}{\left(\Sigma_{V(Y_{ik})0}\right)}^{-1}\left({\tilde{X}}_{iV(Y_{ik})k}-\mu_{V(Y_{ik})0}\right)\right] \end{array}$$

and its penalized version is then

(8) $$\ell_{0}^{\lambda_{0}}\left(\pi_{0},\mu_{0},\Sigma_{0}\right)=\ell_{0}\left(\pi_{0},\mu_{0},\Sigma_{0}\right)-\frac{1}{2}P\left(\lambda_{0},\Sigma_{0}\right).$$

It can be shown, similarly to the unconstrained case, that the expression above is maximized by

(9) $$\begin{array}{cc} {\hat{\pi }}_{0}\left(s\right) & =\frac{s!(p-s)!}{p!}\frac{\sum_{k=1}^{K}\sum_{i=1}^{n_{k}}1\left(|V(Y_{ik})|=s\right)}{n}, \end{array}$$

(10) $$\begin{array}{cc} {\hat{\mu }}_{j0} & =\frac{\sum_{k=1}^{K}\sum_{i=1}^{n_{k}}{\tilde{X}}_{ijk}Y_{ijk}}{\sum_{k=1}^{K}\sum_{i=1}^{n_{k}}Y_{ijk}}, \end{array}$$

(11) $$\begin{array}{cc} {\hat{\Sigma }}_{0}^{\lambda_{0}} & =\frac{\sum_{k=1}^{K}\sum_{i=1}^{n_{k}}\left[Y_{ik}Y_{ik}^{⊤}\circ \left(\left({\tilde{X}}_{ik}-{\hat{\mu }}_{0}\right){\left({\tilde{X}}_{ik}-{\hat{\mu }}_{0}\right)}^{⊤}+\Lambda_{0}\right)\right]}{\sum_{k=1}^{K}\sum_{i=1}^{n_{k}}Y_{ik}Y_{ik}^{⊤}}, \end{array}$$

where $\Lambda_{0}=\mathrm{diag}\left(\lambda_{0}\right)$. In order to test $H_{0}$ we define a test statistic which has the form of a likelihood ratio test (LRT) statistic, given by

(12) $$D^{\lambda ,\lambda_{0}}=-2\left(\ell_{0}\left({\hat{\pi }}_{0},{\hat{\mu }}_{0},{\hat{\Sigma }}_{0}^{\lambda_{0}}\right)-\ell \left(\hat{\pi },\hat{\mu },{\hat{\Sigma }}^{\lambda }\right)\right).$$

When n>p and $\lambda =\lambda_{0}=0$ the expression above reduces to the classical LRT statistic, which Wilks' theorem Wilks (1938) guaranteed to be asymptotically distributed like a Chi‐squared, with degrees of freedom given by the difference in the number of free parameters. This result is not bound to hold in general, therefore we rely on a permutation test (Mielke and Berry 2007; Pesarin 2001), based on the test statistic (12). Namely, we compute the test statistic on the observed data, and on versions in which group labels are permuted uniformly at random. The final p‐value corresponds to the proportion of resamples which yield a larger test statistic than the one observed. The choice of the values of λ and $\lambda_{0}$ used in $D^{\lambda ,\lambda_{0}}$ is discussed in Section 2.3.

### 2.3 Choice of λ and $\lambda_{0}$

The selection of λ and $\lambda_{0}$ can be performed by cross‐validation or by minimizing an information criteria of the form

$$\begin{array}{ccc} IC(\lambda ,\alpha ) & = & \text{goodness of the fit}+\text{penalty}\\ & & \times \;\text{model complexity measure}. \end{array}$$

The second method is faster and what we suggest to use in practice. Measures of model complexity have been extensively studied (Janson et al. 2015). We use the trace of the Fisher information matrix (Bozdogan 1987; Takeuchi 1976), as we detail below.

Let Φ and $\Phi_{0}$ denote the sets of λ and $\lambda_{0}$ that guarantee the positive definiteness of the variance and covariance matrix estimators (7) and (11), respectively. The choice of λ∈Φ is based on the minimization of

(13) $$\begin{array}{cc} \hat{\lambda }=\; & \arg\min_{\lambda \in \Phi }M\left(\lambda ,\hat{\pi },\hat{\mu },\hat{\Sigma }\right)=\arg\min_{\lambda \in \Phi }-2\ell \left(\hat{\pi },\hat{\mu },{\hat{\Sigma }}^{\lambda }\right)\\ & +\left(\log\left(n\right)+\frac{1}{2}\log\left(p\right)\right)\sum_{k=1}^{K}\sum_{i=1}^{n_{k}}\text{tr}\left({\left({\hat{\Sigma }}_{V(Y_{ik})}^{\lambda }\right)}^{-1}\right). \end{array}$$

The expressions $M(\lambda ,\hat{\pi },\hat{\mu },\hat{\Sigma })$ are obtained by reformulating our problem as a weighted regression problem, and using the trace of the Fisher information matrix as a measure of the complexity of the model Bozdogan (1987) (see Appendix B for the complete derivation). In the same spirit, we choose $\lambda_{0}\in \Phi_{0}$ such that

(14) $$\begin{array}{cc} {\hat{\lambda }}_{0}=\; & \arg\min_{\lambda_{0}\in \Phi_{0}}M\left(\lambda_{0},{\hat{\pi }}_{0},{\hat{\mu }}_{0},{\hat{\Sigma }}_{0}\right)\\ =\; & \arg\min_{\lambda_{0}\in \Phi_{0}}-2\ell_{0}\left({\hat{\pi }}_{0},{\hat{\mu }}_{0},{\hat{\Sigma }}_{0}^{\lambda_{0}}\right)+\left(\log\left(n\right)+\frac{1}{2}\log\left(p\right)\right)\\ & \times \sum_{k=1}^{K}\sum_{i=1}^{n_{k}}\text{tr}\left({\left({\hat{\Sigma }}_{0,V(Y_{ik})}^{\lambda_{0}}\right)}^{-1}\right). \end{array}$$

The LRT test statistic (12) is finally denoted as $D^{\hat{\lambda },{\hat{\lambda }}_{0}}$.

## 3 Simulation Study

In this section, we present a simulation study to evaluate the performance of the new test. We consider K=2 and K=4 groups, with balanced sample sizes $n_{1}=\cdots =n_{K}$, with $n_{k}=\left\{5,10\right\}$, and different dimension p={50,100,150,200}. For the variance and covariance matrix we assume the logarithms of the positive observations have unit variance and constant covariance, equal to ρ, with ρ={0,0.4}. We set the mean vector of the first group $\mu_{1}$ equal to the null vector, while the other groups have mean components $\mu_{jk}=\mu_{j1}+c_{1}\left(k-1\right)/\left(K-1\right)$, with $c_{1}=\left\{0,1,5\right\}$, for j=1,⋯,p. The marginal probability of having zeros in the first group is varied as $\pi_{j1}=\left\{0.2,0.5,0.8\right\}$ for j=1,⋯,p, while for the kth group $\pi_{jk}=\pi_{j1}+c_{2}\left(k-1\right)/\left(K-1\right)$, where $c_{2}=\left\{0,0.15,0.3\right\}$, whenever $\pi_{jk}<1$.

Considering all the parameters' combinations we finally evaluate 448 scenarios. For each scenario we generate data 1000 times, we fix a nominal test size at α=0.05, and use 1000 permutations to evaluate the significance level of our proposed test statistic. During the estimation procedure, we additionally choose $\lambda :=\lambda_{1}=\cdots =\lambda_{p}$ and $\lambda_{0}=\lambda_{01}=\cdots =\lambda_{0p}$.

Tables 1, 2, 3 show the proportions of rejections averaged over the 1000 replicates for each scenario with K=2, while Table 4 presents the rejections when K=4. When testing the null hypothesis with two groups, we compare our results to those of Chen et al. (2011). Although Chen's method was not originally intended to address missing data, it is the sole approach in the literature that can be adapted to manage high‐dimensional semicontinuous data. However, this method is limited to two‐group scenarios, meaning the comparison cannot be extended to cases with K=4 groups. Note that for all scenarios in which $c_{1}=c_{2}=0$ the null hypothesis is true, and the proportion of rejections corresponds to the observed test level. In all other cases, the proportion of rejections corresponds to the observed power. The actual size of the regularized MANOVA test closely aligns with the nominal size of 0.05. In some instances, the proportion of rejections slightly exceeds 0.05, but this excess constantly remains below the Monte Carlo error and could be reduced by increasing the number of repetitions. On the other hand, the test proposed by Chen et al. (2011) significantly exceeds the nominal size when the number of observations is small ($n_{k}=5$) and the proportion of zeros is high ($\pi_{j1}=0.8$). This could be because the method was not primarily developed for hypothesis testing in the presence of semicontinuous data. When a large number of components are missing and insufficient information can be recovered from the remaining observations in the sample, it faces difficulties in reaching the correct conclusion. As could be expected the power of the regularized MANOVA test increases with the sample size and with $c_{1}$ and $c_{2}$ growth, which leads to more separation between the two groups. With respect to Chen et al., in the configurations in which the nominal size was guaranteed, our method obtains in general higher or comparable power. The effect of positive correlation among the components leads in the new proposed method to a small decrease in the proportion of rejections. In the cases in which $c_{2}=0$, we additionally observe that higher values of $\pi_{j1}$ lead to lower power. This can be explained by the fact some variables are removed when there is a high probability of zero measurements. Indeed, as described in Section 2.1, some components can be removed during the estimation procedure, resulting on estimates of dimension $p^{*}$, with $p^{*}\leq p$. Tables C1 and C2 in Appendix C report for each scenario the mean over 1000 replicates of $p^{*}$ and of the selected values of λ and $\lambda_{0}$ for K=2 and K=4, respectively. The mean value of $p^{*}$ is notably influenced by the marginal probabilities of missing data in the two groups $\pi_{j1}$ and $\pi_{j2}$. Consequently, we observe a significant decrease in $p^{*}$ when $\pi_{j1}$ and/or $c_{2}$ grow. As expected, when the number of removed variables is too large, the observed power drops significantly. For instance, in the scenario with $\left(K,n_{k},c_{1},c_{2},\pi_{j1},\rho \right)=\left(4,5,0,0.15,0.8,0\right)$ the rejection rate is extremely low. This can be attributed to the very small value of $p^{*}$ in this setting. On the other hand, when the number of observations slightly grows ($n_{k}=10$), our method is able to recover a satisfactory power. Therefore, it can be concluded that, when $p^{*}$ is sufficiently large, the regularized MANOVA test demonstrates good power.

**TABLE 1:** Observed test level for the regularized MANOVA test (scMAN) and for Chen et al. (2011) (Chen) in the presence of semicontinuous high‐dimensional data for K=2 and different values of n, p, $\pi_{j1}$, ρ.

$c_{1}$	$c_{2}$	$\pi_{j1}$	ρ	Test	$n_{k}=5$				$n_{k}=10$			
					p=50	p=100	p=150	p=200	p=50	p=100	p=150	p=200
0	0	0.2	0	scMAN	0.045	0.038	0.058	0.055	0.041	0.058	0.062	0.046
				Chen	0.045	0.046	0.045	0.055	0.035	0.049	0.052	0.058
0	0	0.2	0.4	scMAN	0.048	0.048	0.048	0.053	0.044	0.055	0.043	0.058
				Chen	0.051	0.042	0.046	0.064	0.040	0.045	0.052	0.049
0	0	0.5	0	scMAN	0.046	0.051	0.038	0.043	0.031	0.053	0.047	0.052
				Chen	0.049	0.079	0.033	0.051	0.316	0.080	0.051	0.051
0	0	0.5	0.4	scMAN	0.039	0.043	0.040	0.043	0.042	0.050	0.045	0.053
				Chen	0.050	0.056	0.040	0.056	0.312	0.078	0.049	0.047
0	0	0.8	0	scMAN	0.050	0.051	0.045	0.040	0.048	0.060	0.057	0.050
				Chen	0.756	0.536	0.376	0.245	1.000	1.000	1.000	1.000
0	0	0.8	0.4	scMAN	0.046	0.048	0.047	0.031	0.046	0.055	0.055	0.047
				Chen	0.757	0.533	0.380	0.251	1.000	1.000	1.000	1.000

*Note:* The nominal size is 0.05 and the presented results are the average of 1000 repetitions.

**TABLE 2:** Observed power for the regularized MANOVA test (scMAN) and for Chen et al. (2011) (Chen) in the presence of semicontinuous high‐dimensional data for K=2 and different values of n, p, $c_{1}$, $\pi_{j1}$, ρ.

$c_{1}$	$c_{2}$	$\pi_{j1}$	ρ	Test	$n_{k}=5$				$n_{k}=10$			
					p=50	p=100	p=150	p=200	p=50	p=100	p=150	p=200
1	0	0.2	0	scMAN	0.985	0.994	0.990	0.984	1.000	1.000	1.000	1.000
				Chen	0.851	0.989	0.998	1.000	0.594	0.824	0.935	0.967
1	0	0.2	0.4	scMAN	0.505	0.550	0.529	0.531	0.736	0.837	0.876	0.891
				Chen	0.402	0.481	0.494	0.509	0.382	0.549	0.644	0.702
1	0	0.5	0	scMAN	0.755	0.820	0.714	0.725	0.988	0.959	0.939	0.916
				Chen	0.205	0.268	0.314	0.326	0.323	0.108	0.104	0.133
1	0	0.5	0.4	scMAN	0.420	0.469	0.451	0.443	0.770	0.767	0.769	0.794
				Chen	0.203	0.248	0.295	0.268	0.326	0.114	0.109	0.131
1	0	0.8	0	scMAN	0.134	0.186	0.237	0.219	0.243	0.406	0.548	0.634
				Chen	0.758	0.534	0.392	0.249	1.000	1.000	1.000	1.000
1	0	0.8	0.4	scMAN	0.122	0.161	0.169	0.172	0.250	0.359	0.45	0.514
				Chen	0.758	0.537	0.392	0.258	1.000	1.000	1.000	1.000
5	0	0.2	0	scMAN	1.000	1.000	1.000	1.000	1.000	1.000	1.000	1.000
				Chen	1.000	1.000	1.000	1.000	0.994	1.000	1.000	1.000
5	0	0.2	0.4	scMAN	0.999	0.999	1.000	1.000	1.000	1.000	1.000	1.000
				Chen	1.000	1.000	1.000	1.000	0.995	1.000	1.000	1.000
5	0	0.5	0	scMAN	0.999	1.000	0.999	1.000	1.000	1.000	1.000	1.000
				Chen	0.767	0.837	0.929	0.963	0.327	0.123	0.138	0.199
5	0	0.5	0.4	scMAN	0.999	1.000	0.999	0.988	1.000	1.000	1.000	1.000
				Chen	0.775	0.857	0.944	0.964	0.327	0.123	0.138	0.199
5	0	0.8	0	scMAN	0.688	0.584	0.550	0.521	0.976	0.976	0.987	0.977
				Chen	0.755	0.540	0.401	0.263	1.000	1.000	1.000	1.000
5	0	0.8	0.4	scMAN	0.639	0.556	0.543	0.482	0.972	0.978	0.991	0.985
				Chen	0.755	0.540	0.398	0.264	1.000	1.000	1.000	1.000

*Note:* The nominal size is 0.05 and the presented results are the average of 1000 repetitions.

**TABLE 3:** Observed power for the regularized MANOVA test (scMAN) and for Chen et al. (2011) (Chen) in the presence of semicontinuous high‐dimensional data for K=2 and different values of n, p, $c_{2}$, $\pi_{j1}$, ρ.

$c_{1}$	$c_{2}$	$\pi_{j1}$	ρ	Test	$n_{k}=5$				$n_{k}=10$			
					p=50	p=100	p=150	p=200	p=50	p=100	p=150	p=200
0	0.15	0.2	0	scMAN	0.157	0.132	0.089	0.060	0.546	0.576	0.502	0.426
				Chen	0.079	0.110	0.149	0.164	0.139	0.215	0.278	0.334
0	0.15	0.2	0.4	scMAN	0.107	0.075	0.056	0.059	0.455	0.308	0.168	0.117
				Chen	0.073	0.079	0.089	0.089	0.129	0.174	0.197	0.210
0	0.15	0.5	0	scMAN	0.179	0.100	0.051	0.016	0.360	0.270	0.146	0.084
				Chen	0.093	0.096	0.099	0.125	0.914	0.755	0.589	0.445
0	0.15	0.5	0.4	scMAN	0.149	0.107	0.060	0.043	0.284	0.157	0.078	0.064
				Chen	0.089	0.097	0.102	0.121	0.912	0.755	0.594	0.438
0	0.15	0.8	0	scMAN	0.269	0.379	0.426	0.474	0.936	0.948	0.838	0.671
				Chen	0.994	0.985	0.978	0.965	1.000	1.000	1.000	1.000
0	0.15	0.8	0.4	scMAN	0.261	0.403	0.438	0.473	0.926	0.896	0.778	0.648
				Chen	0.994	0.986	0.977	0.967	1.000	1.000	1.000	1.000
0	0.3	0.2	0	scMAN	0.259	0.139	0.077	0.032	0.853	0.655	0.488	0.414
				Chen	0.304	0.539	0.659	0.777	0.490	0.714	0.842	0.917
0	0.3	0.2	0.4	scMAN	0.170	0.096	0.062	0.050	0.717	0.317	0.146	0.101
				Chen	0.235	0.282	0.331	0.373	0.450	0.648	0.746	0.807
0	0.3	0.5	0	scMAN	0.556	0.303	0.075	0.002	0.824	0.543	0.260	0.148
				Chen	0.273	0.341	0.375	0.438	1.000	0.995	0.991	0.988
0	0.3	0.5	0.4	scMAN	0.491	0.330	0.142	0.040	0.694	0.348	0.164	0.113
				Chen	0.258	0.333	0.373	0.406	1.000	0.995	0.990	0.987

*Note:* The nominal size is 0.05 and the presented results are the average of 1000 repetitions.

**TABLE 4:** Proportion of rejections for the regularized MANOVA test for semicontinuous high‐dimensional data for K=4 and different values of n, p, $c_{1}$, $c_{2}$, $\pi_{j1}$, ρ.

$c_{1}$	$c_{2}$	$\pi_{j1}$	ρ	p=50	$n_{k}=5$				$n_{k}=10$		
					p=100	p=150	p=200	p=50	p=100	p=150	p=200
0	0	0.2	0	0.062	0.067	0.066	0.066	0.050	0.042	0.046	0.053
0	0	0.2	0.4	0.056	0.055	0.057	0.055	0.048	0.040	0.061	0.053
0	0	0.5	0	0.041	0.050	0.054	0.050	0.060	0.055	0.049	0.058
0	0	0.5	0.4	0.045	0.037	0.048	0.039	0.067	0.055	0.055	0.059
0	0	0.8	0	0.036	0.042	0.048	0.065	0.053	0.053	0.055	0.061
0	0	0.8	0.4	0.037	0.048	0.042	0.058	0.046	0.049	0.049	0.047
1	0	0.2	0	0.845	0.991	1.000	1.000	0.998	1.000	1.000	1.000
1	0	0.2	0.4	0.349	0.408	0.444	0.481	0.546	0.692	0.760	0.770
1	0	0.5	0	0.487	0.781	0.846	0.860	0.941	1.000	1.000	1.000
1	0	0.5	0.4	0.271	0.334	0.325	0.356	0.615	0.764	0.836	0.816
1	0	0.8	0	0.059	0.084	0.102	0.127	0.146	0.247	0.355	0.436
1	0	0.8	0.4	0.056	0.084	0.086	0.092	0.147	0.228	0.281	0.343
5	0	0.2	0	1.000	0.991	0.984	0.996	1.000	1.000	1.000	1.000
5	0	0.2	0.4	1.000	0.992	0.982	0.998	1.000	1.000	1.000	1.000
5	0	0.5	0	0.881	0.977	0.996	1.000	0.993	1.000	1.000	1.000
5	0	0.5	0.4	0.848	0.946	0.976	0.988	0.992	1.000	0.999	1.000
5	0	0.8	0	0.246	0.340	0.361	0.374	0.894	0.952	0.985	0.984
5	0	0.8	0.4	0.225	0.308	0.329	0.333	0.857	0.925	0.965	0.967
0	0.15	0.2	0	0.157	0.224	0.215	0.225	0.400	0.486	0.509	0.526
0	0.15	0.2	0.4	0.129	0.115	0.122	0.088	0.357	0.370	0.339	0.248
0	0.15	0.5	0	0.129	0.153	0.138	0.141	0.320	0.394	0.341	0.317
0	0.15	0.5	0.4	0.115	0.095	0.08	0.067	0.274	0.237	0.205	0.134
0	0.15	0.8	0	0.000	0.006	0.014	0.040	0.712	0.901	0.903	0.822
0	0.15	0.8	0.4	0.000	0.006	0.019	0.036	0.685	0.859	0.843	0.763
0	0.3	0.2	0	0.343	0.438	0.460	0.486	0.852	0.867	0.818	0.796
0	0.3	0.2	0.4	0.266	0.217	0.184	0.156	0.814	0.708	0.498	0.348
0	0.3	0.5	0	0.474	0.543	0.484	0.367	0.917	0.930	0.881	0.796
0	0.3	0.5	0.4	0.439	0.400	0.318	0.219	0.862	0.755	0.515	0.313

*Note:* The nominal size is 0.05 and the presented results are the average of 1000 repetitions.

We simulate also two additional scenarios that mimic the real example described in Section 4.1. Hence, we test a situation with $n_{1}=n_{2}=5$, p=339, both under the null (scenario A) and under the alternative hypothesis (scenario B). The mean of the normal distribution used to simulate the data in scenarios A and B is equal to the sample mean of the blastocyst data presented in Section 4.1 under the null and under the alternative hypothesis respectively. In the second case, some of the components of the sample means of the different groups were missing, therefore we imputed the missing values with the corresponding components under $H_{0}$. In a similar vein, the marginal probability of a missing component is equal to its sample version in the two different scenarios. Also, the sample variance and covariance matrices had some missing entries. Hence, we have imputed the missing elements along the main diagonal with 1 and the ones that were not on the main diagonal with 0. To achieve the positive definiteness, we additionally added 0.1 to each element on the main diagonal. The proportion of rejections in scenario A is equal to 0.051, while under scenario B it is 0.874. The mean dimension of components used for the estimation is respectively 251.74 and 261.01.

## 4 Real‐Data Analyses

We describe in this section the analysis of two real‐data examples.

### 4.1 MicroRNA Differential Expression in Blastocyst Cultures

The study of microRNA expression is particularly useful for the assessment of biological pathways, especially in the early‐stage development (Capalbo et al. 2016). In our original data example, we have n=10 samples coming from human blastocysts (day 5 after in vitro fertilization). Simplifying the setting, it can be said that blastocysts are composed by about 120 cells, about one‐third of which differentiated into an inner cell mass (ICM), and the remaining forming an outer TrophoEctoderm (TE). The ICM will proceed to develop into a human being, and the TE will develop into a placenta and other annexes.

In our data, we have $n_{1}=5$ samples from ICM and $n_{2}=5$ samples from TE. MicroRNA expression of p=339 sequences was obtained from each sample, with technical details that can be found elsewhere (Capalbo et al. 2016). MicroRNA are short sequences that are known to coordinate the expression of pathways of genes. Since many pathways are not yet activated at the blastocyst stage, many microRNA sequences will not be found in the samples, leading to zero‐inflation. This is confirmed in our data, where only about 39% of the sequences are non‐zero in all samples.

The main idea is to test the biological hypothesis that even if cells are not drastically differentiated at the blastocyst stage, their microRNA expression already is showing different pathway activation. In our framework and notation, this precisely would correspond to rejecting

(15) $$H_{0}:\left(\cap_{s=0}^{p}\pi_{1}\left(s\right)=\pi_{2}\left(s\right)\right)\cap \left(\cap_{j=1}^{p}\mu_{j1}=\mu_{j2}\right)$$

indicating that there exist at least one microRNA that is expressed with differential probability or level of expression when comparing ICM with trophectoderm. Due to the very large number of zeros in the data it is clear that any test assuming multivariate normality, without taking into account the probability masses at zero, would be biased even if well calibrated for the p>n setting.

We use our proposed test to verify (15), and obtain a test statistic of 15.15 and, after permutation based on 1000 replicates, the *p*‐value is 0.008. We then reject the null hypothesis and conclude that there is an overall difference in microRNA expression levels when comparing ICM and TE at day 5 after fertilization. Interestingly, 53.4% of microRNA measurements are zero in the ICM, while 35.7% in the TE, which indicates an higher level of the biological activity for the TE. Similarly, 80.8% estimated mean expressions are larger for TE than for ICM.

### 4.2 Alien Species Invasion in Socotra Island

Socotra is an island located in the Arabian sea and administratively belonging to Yemen. Its rich biodiversity and the presence of many endemic species make it a unique environment (e.g., Attorre et al. 2014; Riccardi et al. 2020). Socotra environment is endangered by many threats, including climate change, anthropic activities, and alien species invasion. In this section, we use an original dataset about the determinants of alien species invasion in the island. See Terzano et al. (2018) for a similar assessment related to South Africa.

Researchers explored the island to record the abundance of p=299 alien species in n=103 plots that were approximately square shaped with an area of about 0.5 ha each. The abundance measure was the widely used importance value (IV), which can be calculated as

$$\text{IV}\left(s\right)=100\frac{\text{NS}(s)}{\sum_{u}\text{NS}\left(u\right)}+100\frac{\text{BA}(s)}{\sum_{u}\text{BA}\left(u\right)},$$

where NS(s) denotes the number of stems for species s that were counted in the plot, and BA(s) the basal area occupied by species s as the sum of areas occupied at breast height by each individual stem. IV ranges then from 0 (species is absent) to 200 (the plot is entirely covered only by the species). Despite the species considered being potentially very invasive, they are (still) absent in many plots, with about 93% of entries being equal to zero.

Plots can be classified as being limestone, alluvial, or granite. Our main question involves the assessment of association of overall abundance of invasive alien species and the geological characteristics of the plots. We conducted a comparison across the three types of plots, yielding a *p*‐value of 0.04 and a test statistic of 139.427. As a result, we reject the null hypothesis, concluding that there is a significant overall difference in the distribution of alien species across the different plot types.

## 5 Conclusions

In this paper, we have presented, to the best of our knowledge, the first MANOVA test for high‐dimensional semicontinous data. The test depends on a penalty parameter, which can be chosen in different ways. Importantly, the permutation procedure used to compute the significance level allows the user to naturally take into account additional heterogeneity arising from any data‐driven penalty parameter choice. The derivation of the formal asymptotic distribution, especially for unknown λ, would be very cumbersome (Han and Yin 2023). A limitation of the permutation approach is, clearly, its computational complexity. We shall report however that our implementation is rather efficient, both thanks to the closed form solution for the MLE and the use of parallel computing. A future contribution might go beyond the parametric assumptions (Zhang and Feng 2024), maybe based on a test statistic that involves the estimated traces of the covariance matrices (Chen and Qin 2010; Hu et al. 2017; Srivastava and Kubokawa 2013; Yamada and Himeno 2015).

## Software

Software in the form of R package semicontMANOVA is available on the publisher's website and on CRAN.

## Conflicts of Interest

The authors declare no conflicts of interest.

### Open Research Badges

This article has earned an Open Data badge for making publicly available the digitally‐shareable data necessary to reproduce the reported results. The data is available in the Supporting Information section.

This article has earned an open data badge “**Reproducible Research**” for making publicly available the code necessary to reproduce the reported results. The results reported in this article could fully be reproduced.

## Supporting information

Supporting information

## Data Availability

R codes to implement our proposed methods and to replicate our simulation studies are provided as a supplement to this paper and in the R package semicontMANOVA. The data that support the findings of this study are not available and are not publicly available due to privacy.

## Appendix A Estimation of Parameters

We want to derive ${\hat{\pi }}_{k}\left(s\right)$, ${\hat{\mu }}_{k}$ and ${\hat{\Sigma }}^{\lambda }$, with k=1,⋯,K, s=0,⋯,p, that maximize the regularized log‐likelihood (3).

### A.1 Estimation of the Parameters of the Discrete Part

First of all, we derive estimators of the discrete part. The constraint on the sum of the Bernoulli parameters $\sum_{a\in {\left\{0,1\right\}}^{p}}\pi_{k}\left(a\right)=1$ can be rewritten, using the homogeneity condition (1), obtaining

(A1)
∑s=0ppsπk(s)=1for a fixed k∈{1,⋯,K},

where the binomial coefficient is the number of $a\in {\left\{0,1\right\}}^{p}$ with s components equal to 1. Fixing r≠0, we take derivative of the regularized log‐likelihood (3), with respect to $\pi_{k}\left(r\right)$

(A2)
$$\begin{array}{cc} & \frac{\partial \ell^{\lambda }\left(\pi ,\mu ,\Sigma \right)}{\partial \pi_{k}\left(r\right)}\\ & \;=\sum_{i=1}^{n_{k}}\frac{-\frac{p!}{r!(p-r)!}1\left(|V(Y_{ik})|=0\right)\pi_{k}\left(r\right)+1\left(|V(Y_{ik})|=r\right)\pi_{k}\left(0\right)}{\left(1-\sum_{s=1}^{p}\frac{p!}{s!(p-s)!}\pi_{k}\left(s\right)\right)\pi_{k}\left(r\right)}. \end{array}$$

The expression above is equal to zero, using (A1), if

(A3)
$${\hat{\pi }}_{k}\left(r\right)={\hat{\pi }}_{k}\left(0\right)\frac{r!(p-r)!}{p!}\frac{\sum_{i=1}^{n_{k}}1\left(|V(Y_{ik})|=r\right)}{\sum_{i=1}^{n_{k}}1\left(|V(Y_{ik})|=0\right)}$$

for each r=1,⋯,p. Using (A1), we also obtain

$${\hat{\pi }}_{k}\left(0\right)=\frac{\sum_{i=1}^{n_{k}}1\left(|V(Y_{ik})|=0\right)}{\sum_{s=0}^{p}\sum_{i=1}^{n_{k}}1\left(|V(Y_{ik})|=s\right)}=\frac{\sum_{i=1}^{n_{k}}1\left(|V(Y_{ik})|=0\right)}{n_{k}}$$

and we can conclude that the estimator for the discrete part is

(A4)
$${\hat{\pi }}_{k}\left(r\right)=\frac{r!(p-r)!}{p!}\frac{\sum_{i=1}^{n_{k}}1\left(|V(Y_{ik})|=r\right)}{n_{k}}\;\text{for}\;r=0,\cdots ,p.$$

The derivation of the estimator under the null hypothesis follows the same reasoning after defining $\pi_{0}\left(s\right):=\pi_{1}\left(s\right)=\cdots =\pi_{K}\left(s\right)$.

### A.2 Estimation of Parameters for the Continuous Part

In the expression of the likelihood $\mu_{k}$ appears as subsets $\mu_{V(Y_{ik})}$, with $i=1,\cdots ,n_{k}$. Note that under model specification we can separate the presence/absence and continuous parts of the likelihood. We have

(A5)
$$\frac{\partial \ell^{\lambda }\left(\pi ,\mu ,\Sigma \right)}{\partial \mu_{V(Y_{ik})k}}={\left(\Sigma_{V(Y_{ik})}\right)}^{-1}\left({\tilde{X}}_{iV(Y_{ik})k}-\mu_{V(Y_{ik})k}\right).$$

The expression above is equal to zero as soon as ${\hat{\mu }}_{V(Y_{ik})k}={\tilde{X}}_{iV(Y_{ik})k}$, that implies

(A6)
$$\left(\begin{array}{c} {\hat{\mu }}_{1k}Y_{i1k}\\ \vdots \\ {\hat{\mu }}_{pk}Y_{ipk} \end{array}\right)=\left(\begin{array}{c} {\tilde{X}}_{i1k}Y_{i1k}\\ \vdots \\ {\tilde{X}}_{ipk}Y_{ipk}, \end{array}\right)$$

where ${\tilde{X}}_{ik}$ corresponds to the vector of $R^{p}$ with elements

$${\tilde{X}}_{ijk}=\left\{\begin{array}{cc} \log(X_{ijk}) & \;\text{if}\;Y_{ijk}=1,\\ 0 & \;\text{if}\;Y_{ijk}=0. \end{array}\right.$$

Equality (A6) holds for each $i\in \{1,\cdots ,n_{k}\}$, hence we easily obtain

(A7)
$${\hat{\mu }}_{jk}=\frac{\sum_{i=1}^{n_{k}}{\tilde{X}}_{ijk}Y_{ijk}}{\sum_{i=1}^{n_{k}}Y_{ijk}}\;\text{for}\;k=1,\cdots ,K;\;j=1,\cdots ,p,$$

that coincides with the MLE (Farcomeni 2016). In the same way, we can derive the estimator under the null hypothesis ${\hat{\mu }}_{0}$, shown in Equation (10).

We now discuss ${\hat{\Sigma }}^{\lambda }$. We simplify the notation, indicating $\Sigma_{V}$ in place of $\Sigma_{V(Y_{ik})}$. For fixed i and k we differentiate the log‐likelihood with respect to $\Sigma_{V}$. Exploiting the symmetry of $\Sigma_{V}$ (Petersen et al. 2008), we obtain

(A8)
$$\begin{array}{ccc} \frac{\partial \ell^{\lambda }\left(\pi ,\mu ,\Sigma \right)}{\partial \Sigma_{V}} & = & -\frac{1}{2}\left[2\Sigma_{V}^{-1}-\Sigma_{V}^{-1}\circ I_{V}\right]-\frac{1}{2}\left[-2\Sigma_{V}^{-1}\left({\tilde{X}}_{V}-\mu_{V}\right){\left({\tilde{X}}_{V}-\mu_{V}\right)}^{⊤}\Sigma_{V}^{-1}\right.\\ & & \left.+\Sigma_{V}^{-1}\left({\tilde{X}}_{V}-\mu_{V}\right){\left({\tilde{X}}_{V}-\mu_{V}\right)}^{⊤}\Sigma_{V}^{-1}\circ I_{V}\right]\\ & & -\frac{1}{2}\left[-2\Sigma_{V}^{-1}\Lambda_{V}\Sigma_{V}^{-1}+\Sigma_{V}^{-1}\Lambda_{V}\Sigma_{V}^{-1}\circ I_{V}\right]. \end{array}$$

The expression above is equal to zero when we set

(A9)
$$\begin{array}{c} {\left({\hat{\Sigma }}_{V}\right)}^{-1}={\left({\hat{\Sigma }}_{V}\right)}^{-1}\left({\tilde{X}}_{V}-\mu_{V}\right){\left({\tilde{X}}_{V}-\mu_{V}\right)}^{⊤}{\left({\hat{\Sigma }}_{V}\right)}^{-1}+{\left({\hat{\Sigma }}_{V}\right)}^{-1}\Lambda_{V}{\left({\hat{\Sigma }}_{V}\right)}^{-1}, \end{array}$$

that implies

(A10)
$${\hat{\Sigma }}_{V}^{\lambda }=\left({\tilde{X}}_{V}-\mu_{V}\right){\left({\tilde{X}}_{V}-\mu_{V}\right)}^{⊤}+\Lambda_{V},$$

where we have added the apex λ to underline that the estimator depends on the parameter λ. As before we consider the full p×p matrix, obtained summing over groups and observations, i.e.,

(A11)
$$\begin{array}{c} {\hat{\Sigma }}^{\lambda }\circ \sum_{k=1}^{K}\sum_{i=1}^{n_{k}}\left(Y_{ik}Y_{ik}^{⊤}\right)=\sum_{k=1}^{K}\sum_{i=1}^{n_{k}}\left[\left(Y_{ik}Y_{ik}^{⊤}\right)\circ \left(\left({\tilde{X}}_{ik}-{\hat{\mu }}_{k}\right){\left({\tilde{X}}_{ik}-{\hat{\mu }}_{k}\right)}^{⊤}+\Lambda \right)\right]. \end{array}$$

From (A11), we directly derive the final regularized estimator for the variance and covariance matrix (7). The estimator (11) under the null hypothesis is computed with similar reasoning.

## Appendix B Details on the Selection of Penalty Parameters

Let $n_{0}:=0$ and $n_{k}^{*}=\sum_{t=0}^{k-1}n_{t}$ be the sample size considering the observations in the first (k−1)‐th groups, with k=1,⋯,K. We define the function $h:N^{2}\to N$ that associates with each couple (i,k) a unique scalar l such that $l=h\left(i,k\right):=i+n_{k}^{*}$. It is straightforward to check that l=1,⋯,n and, for fixed k, $n_{k}^{*}+1\leq h\left(i,k\right)\leq n_{k+1}^{*}$. Let $Z_{l}:={\tilde{X}}_{ik}|Y_{ik}$ be the $p_{l}$‐dimensional vector, where $p_{l}:=\sum_{j=1}^{p}Y_{ijk}$ is the number of positive components of $Y_{ik}$. In the following discussion, we will use l and (i,k) in an interchangeable way, to denote the specific configuration of i and k such that l=h(i,k).

The assumption of conditional Gaussian distribution (2) leads to $Z_{l}∼N_{p_{l}}\left(\mu_{l},\Sigma_{l}\right)$, with $\mu_{l}:=\mu_{V(Y_{ik})k}$ and $\Sigma_{l}=\Sigma_{V\left(Y_{ik}\right)}$. Equivalently, we can write $Z_{l}$ as

$$Z_{l}=I_{p_{l}}\mu_{l}+\varepsilon_{l},$$

where $I_{p_{l}}$ is a $p_{l}\times p_{l}$ identity matrix, $\varepsilon_{l}∼N_{p_{l}}\left(0,\Sigma_{l}\right)$ and $\varepsilon_{l}⊥\;\;\;⊥\varepsilon_{r}$ for l≠r. Stacking $Z_{l}$ together we define

(B1)
$$Z=\left(\begin{array}{c} Z_{1}\\ \vdots \\ Z_{n} \end{array}\right)=X\mu +\varepsilon ,$$

where X is a $\left(\sum_{l=1}^{n}p_{l}\right)\times \left(\sum_{l=1}^{n}p_{l}\right)$‐dimensional matrix containing the regression coefficients, i.e.,

(B2)
$$X=\left(\begin{array}{ccccc} I_{p_{1}} & 0 & 0 & \cdots & 0\\ 0 & I_{p_{2}} & 0 & \ddots & 0\\ 0 & 0 & \ddots & \ddots & 0\\ \vdots & \ddots & \ddots & \ddots & 0\\ 0 & 0 & \cdots & 0 & I_{p_{n}} \end{array}\right),$$

while $\mu ={\left(\mu_{1},\;\mu_{2},\;\cdots ,\;\mu_{n}\right)}^{⊤}$ and $\varepsilon ={\left(\varepsilon_{1},\;\varepsilon_{2},\;\cdots ,\;\varepsilon_{n}\right)}^{⊤}$. Consequently, $\varepsilon ∼N_{\sum_{l=1}^{n}p_{l}}\left(0,\Omega \right)$, where

(B3)
$$\Omega =\left(\begin{array}{ccccccc} \Sigma_{V(Y_{11})} & & 0 & & \cdots & & 0\\ & \ddots & & & & & \\ 0 & & \Sigma_{V(Y_{n_{1}1})} & & \ddots & & \vdots \\ & & & \ddots & & & \\ \vdots & & \ddots & & \Sigma_{V(Y_{1n_{K}})} & & 0\\ & & & & & \ddots & \\ 0 & & \cdots & & 0 & & \Sigma_{V(Y_{n_{K}K})} \end{array}\right).$$

Hence, (B1) corresponds to rewriting the problem as a weighted linear regression, with weights equal to the inverse of the variance and covariance matrix Ω. The Fisher information matrix of the problem is

$$I=X^{⊤}\Omega^{-1}X=\Omega^{-1},$$

and its trace can be used as a measure of the complexity of the model (Takeuchi 1976; Bozdogan 1987; Boisbunon et al. 2014). Since Ω is a diagonal matrix, we have

(B4)
$$\text{tr}\left(I\right)=\text{tr}\left(\Omega^{-1}\right)=\sum_{k=1}^{K}\sum_{i=1}^{n_{k}}\text{tr}\left(\Sigma_{V(Y_{ik})}^{-1}\right).$$

We mimick the form of the Akaike information criterion (AIC) and define the quantity $M(\lambda ,\hat{\pi },\hat{\mu },\hat{\Sigma })$

(B5)
$$\begin{array}{ccc} M(\lambda ,\hat{\pi },\hat{\mu },\hat{\Sigma }): & = & -2\log\left(L^{\lambda }\left(\hat{\pi },\hat{\mu },{\hat{\Sigma }}^{\lambda }\right)\right)\\ & & +\left(\log\left(n\right)+\frac{1}{2}\log\left(p\right)\right)\sum_{k=1}^{K}\sum_{i=1}^{n_{k}}\text{tr}\left({\left({\hat{\Sigma }}_{V(Y_{ik})}^{\lambda }\right)}^{-1}\right).\; \end{array}$$

This quantity combines the goodness of the model and its complexity, therefore it is used to select $\hat{\lambda }$, as shown in (13).

## Appendix C Additional Tables

**TABLE C1:** Each entrance of the table contains the mean dimension $p^{*}$ of the variance and covariance matrix estimate and the mean values of λ and $\lambda_{0}$ for different values of n, p, $c_{1}$, $c_{2}$, $\pi_{j1}$, ρ for K=2.

$c_{1}$	$c_{2}$	$\pi_{j1}$	ρ	$n_{k}=5$				$n_{k}=10$			
				p=50	p=100	p=150	p=200	p=50	p=100	p=150	p=200
0	0	0.2	0	49.93; 4.37–4.41	99.82; 4.78–4.84	149.66; 5.03–5.10	199.50; 5.21–5.30	50.00; 5.01–5.01	100.00; 5.41–5.42	150; 5.65–5.66	200.00; 5.82–5.83
0	0	0.2	0.4	49.93; 4.39–4.44	99.82; 4.83–4.92	149.66; 5.12–5.30	199.50; 5.46–5.78	50.00; 5.03–5.04	10.000; 5.43–5.44	150; 5.67–5.68	200.00; 5.84–5.86
0	0	0.5	0	37.22; 4.36–4.59	69.50; 4.96–5.53	100.75; 5.49–6.42	130.81; 6.10–7.45	47.90; 5.16–5.23	93.54; 5.75–5.92	137.87; 6.13–6.4	181.67; 6.5–6.93
0	0	0.5	0.4	37.22; 4.46–4.84	69.50; 5.22–6.12	100.75; 6.07–7.61	130.81; 7.09–9.13	47.90; 5.26–5.39	93.54; 5.98–6.41	137.87; 6.74–7.49	181.67; 7.63–8.71
0	0	0.8	0	7.99; 3.35–3.42	13.61; 3.66–3.82	19.09; 3.87–4.15	24.26; 4.02–4.41	13.38; 4.38–4.48	21.47; 4.70–4.92	27.68; 4.92–5.24	33.91; 5.12–5.53
0	0	0.8	0.4	7.99; 3.36–3.45	13.61; 3.67–3.86	19.09; 3.90–4.23	24.26; 4.06–4.51	13.38; 4.42–4.60	21.47; 4.78–5.07	27.68; 5.03–5.46	33.91; 5.23–5.74
1	0	0.2	0	49.93; 4.37–4.49	99.82; 4.78–4.98	149.66; 5.03–5.31	199.50; 5.21–5.64	50.00; 5.01–5.06	100.00; 5.41–5.49	150; 5.65–5.74	200.00; 5.82–5.92
1	0	0.2	0.4	49.93; 4.39–4.58	99.82; 4.83–5.25	149.66; 5.12–6.02	199.50; 5.46–7.06	50.00; 5.03–5.09	100.00; 5.43–5.52	150; 5.67–5.78	200.00; 5.84–5.99
1	0	0.5	0	37.22; 4.36–5.00	69.50; 4.96–6.66	100.75; 5.49–8.08	130.81; 6.10–9.57	47.90; 5.16–5.48	93.54; 5.75–6.53	137.87; 6.13–7.55	181.67; 6.5–8.69
1	0	0.5	0.4	37.22; 4.46–5.61	69.50; 5.22–7.91	100.75; 6.07–10.05	130.81; 7.09–12.42	47.90; 5.26–5.89	93.54; 5.98–7.82	137.87; 6.74–9.8	181.67; 7.63–11.83
1	0	0.8	0	7.99; 3.35–3.51	13.61; 3.66–4.01	19.09; 3.87–4.50	24.26; 4.02–4.92	13.38; 4.38–4.64	21.47; 4.70–5.30	27.68; 4.92–5.76	33.91; 5.12–6.28
1	0	0.8	0.4	7.99; 3.36–3.59	13.61; 3.67–4.10	19.09; 3.90–4.65	24.26; 4.06–5.13	13.38; 4.42–6.00	21.47; 4.78–5.61	27.68; 5.03–6.17	33.91; 5.23–6.72
5	0	0.2	0	49.93; 4.37–10.26	99.82; 4.78–18.56	149.66; 5.03–26.91	199.50; 5.21–35.30	50.00; 5.01–6.85	100.00; 5.41–9.83	150; 5.65–13.89	200.00; 5.82–18.07
5	0	0.2	0.4	49.93; 4.39–11.20	99.82; 4.83–20.72	149.66; 5.12–29.94	199.50; 5.46–39.25	50.00; 5.03–7.04	100.00; 5.43–10.66	150; 5.67–15.22	200.00; 5.84–19.75
5	0	0.5	0	37.22; 4.36–19.07	69.50; 4.96–31.31	100.75; 5.49–42.12	130.81; 6.10–53.13	47.90; 5.16–18.04	93.54; 5.75–29.97	137.87; 6.13–41.02	181.67; 6.5–52.18
5	0	0.5	0.4	37.22; 4.46–21.71	69.50; 5.22–36.12	100.75; 6.07–48.70	130.81; 7.07–61.79	47.90; 5.26–20.68	93.54; 5.98–34.64	137.87; 6.74–47.27	181.67; 7.63–60.01
5	0	0.8	0	7.99; 3.35–7.62	13.61; 3.66–11.06	19.09; 3.87–14.46	24.26; 4.02–17.14	13.38; 4.38–12.82	21.47; 4.70–17.65	27.68; 4.92–20.76	33.91; 5.12–23.91
5	0	0.8	0.4	7.99; 3.36–7.89	13.61; 3.67–11.5	19.09; 3.90–15.04	24.26; 4.06–18.06	13.38; 4.42–13.73	21.47; 4.78–19.05	27.68; 5.03–22.34	33.91; 5.23–25.85
0	0.15	0.2	0	49.43; 4.42–4.48	98.56; 4.87–4.98	147.47; 5.17–5.35	196.35; 5.42–5.70	49.99; 5.04–5.05	100.00; 5.46–5.48	149.99; 5.72–5.74	199.99; 5.90–5.93
0	0.15	0.2	0.4	49.43; 4.46–4.55	98.56; 4.99–5.21	147.47; 5.48–5.95	196.35; 6.15–6.93	49.99; 5.07–5.08	100.00; 5.49–5.51	149.99; 5.75–5.79	199.99; 5.95–6.00
0	0.15	0.5	0	29.55; 4.23–4.43	54.59; 4.86–5.32	78.28; 5.55–6.30	101.42; 6.33–7.27	43.06; 5.16–5.25	81.86; 5.75–5.98	118.39; 6.23–6.64	154.1; 6.75–7.30
0	0.15	0.5	0.4	29.55; 4.32–4.62	54.59; 5.09–5.81	78.28; 5.90–7.05	101.42; 6.94–8.48	43.06; 5.30–5.48	81.86; 6.12–6.62	118.39; 7.04–7.87	154.1; 7.98–9.10
0	0.15	0.8	0	2.29; 1.97–1.97	3.70; 2.72–2.74	4.86; 3.01–3.03	6.39; 3.22–3.25	5.01; 3.75–3.76	8.76; 4.13–4.16	12.25; 4.34–4.39	15.79; 4.53–4.62
0	0.15	0.8	0.4	2.29; 1.97–1.98	3.70; 2.73–2.75	4.86; 3.02–3.04	6.39; 3.22–3.26	5.01; 3.75–3.77	8.76; 4.14–4.20	12.25; 4.38–4.46	15.79; 4.58–4.70
0	0.3	0.2	0	47.7; 4.44–4.52	94.62; 4.96–5.12	141.32; 5.37–5.65	187.8; 5.79–6.21	49.94; 5.06–5.07	99.89; 5.51–5.53	149.82; 5.78–5.81	199.77; 5.97–6.01
0	0.3	0.2	0.4	47.7; 4.51–4.63	94.62; 5.22–5.53	141.32; 5.92–6.48	187.8; 6.85–7.66	49.94; 5.10–5.11	99.89; 5.55–5.58	149.82; 5.84–5.89	199.77; 6.10–6.17
0	0.3	0.5	0	20.72; 4.00–4.12	38.71; 4.62–4.89	56.55; 5.28–5.76	74.18; 6.12–6.69	35.58; 5.07–5.14	66.92; 5.69–5.84	96.03; 6.23–6.51	124.39; 6.84–7.21
0	0.3	0.5	0.4	20.72; 4.08–4.23	38.71; 4.80–5.17	56.55; 5.49–6.10	74.18; 6.43–7.30	35.58; 5.23–5.34	66.92; 6.09–6.39	96.03; 7.04–7.55	124.39; 7.95–8.61

*Note:* The presented results are the average of 1000 repetitions.

**TABLE C2:** Each entrance of the table contains the mean dimension $p^{*}$ of the variance and covariance matrix estimate and the mean values of λ and $\lambda_{0}$ for different values of n, p, $c_{1}$, $c_{2}$, $\pi_{j1}$, ρ for K=4.

$c_{1}$	$c_{2}$	$\pi_{j1}$	ρ	$n_{k}=5$				$n_{k}=10$			
				p=50	p=100	p=150	p=200	p=50	p=100	p=150	p=200
0	0	0.2	0	49.94; 5.00–5.01	99.87; 5.39–5.42	149.80; 5.62–5.66	199.74; 5.79–5.83	50.00; 5.66–5.67	100.00; 6.04–6.04	150.00; 6.27–6.27	200.00; 6.43–6.44
0	0	0.2	0.4	49.94; 5.01–5.04	99.87; 5.40–5.44	149.80; 5.63–5.68	199.74; 5.80–5.86	50.00; 5.68–5.69	100; 6.06–6.07	150; 6.28–6.30	200.00; 6.45–6.46
0	0	0.5	0	42.79; 4.98–5.12	84.38; 5.44–5.72	124.98; 5.74–6.17	165.29; 5.96–6.53	49.76; 5.72–5.75	99.57; 6.18–6.25	149.33; 6.49–6.59	199.05; 6.72–6.84
0	0	0.5	0.4	42.79; 5.03–5.24	84.38; 5.50–6.03	124.98; 5.84–6.91	165.29; 6.15–7.83	49.76; 5.78–5.83	99.57; 6.25–6.35	149.33; 6.55–6.70	199.05; 6.80–7.00
0	0	0.8	0	5.38; 3.74–3.78	9.33; 4.10–4.18	12.65; 4.27–4.41	15.87; 4.39–4.57	16.29; 5.12–5.23	26.61; 5.41–5.58	35.56; 5.59–5.85	43.18; 5.71–6.07
0	0	0.8	0.4	5.38; 3.75–3.82	9.33; 4.11–4.26	12.65; 4.28–4.49	15.87; 4.40–4.65	16.29; 5.15–5.35	26.61; 5.46–5.86	35.56; 5.66–6.25	43.18; 5.81–6.61
1	0	0.2	0	49.94; 5.00–5.04	99.87; 5.39–5.46	149.8; 5.62–5.70	199.74; 5.79–5.88	50.00; 5.66–5.68	100.00; 6.04–6.07	150.00; 6.27–6.30	200.00; 6.43–6.48
1	0	0.2	0.4	49.94; 5.01–5.07	99.87; 5.40–5.48	149.8; 5.63–5.74	199.74; 5.80–5.93	50.00; 5.68–5.71	100.00; 6.06–6.09	150.00; 6.28–6.33	200.00; 6.45–6.5.
1	0	0.5	0	42.79; 4.98–5.22	84.38; 5.44–5.94	124.98; 5.74–6.56	165.29; 5.96–7.18	49.76; 5.72–5.81	99.57; 6.18–6.35	149.33; 6.49–6.71	199.05; 6.72–6.99
1	0	0.5	0.4	42.79; 5.03–5.45	84.38; 5.50–6.60	124.98; 5.84–7.95	165.29; 6.15–9.46	49.76; 5.78–5.91	99.57; 6.25–6.49	149.33; 6.55–6.90	199.05; 6.80–7.39
1	0	0.8	0	5.38; 3.74–3.80	9.33; 4.10–4.24	12.65; 4.27–4.49	15.87; 4.39–4.69	16.29; 5.12–5.31	26.61; 5.41–5.78	35.56; 5.59–6.14	43.18; 5.71–6.48
1	0	0.8	0.4	5.38; 3.75–3.87	9.33; 4.11–4.37	12.65; 4.28–4.62	15.87; 4.40–4.85	16.29; 5.15–5.52	26.61; 5.46–6.28	35.56; 5.66–6.86	43.18; 5.81–7.36
5	0	0.2	0	49.94; 5–6.24	99.87; 5.39–8.20	149.80; 5.62–10.85	199.74; 5.79–13.82	50.00; 5.66–6.24	100.00; 6.04–6.99	150.00; 6.27–7.61	200.00; 6.43–8.27
5	0	0.2	0.4	49.94; 5.01–6.35	99.87; 5.40–8.66	149.80; 5.63–11.61	199.74; 5.80–14.88	50.00; 5.68–6.24	100.00; 6.06–7.01	150.00; 6.28–7.72	200.00; 6.45–8.55
5	0	0.5	0	42.79; 4.98–14.81	84.38; 5.44–24.16	124.98; 5.74–32.34	165.29; 5.96–40.26	49.76; 5.72–10.28	99.57; 6.18–17.01	149.33; 6.49–22.83	199.05; 6.72–28.37
5	0	0.5	0.4	42.79; 5.03–15.92	84.38; 5.5–26.36	124.98; 5.84–35.23	165.29; 6.15–44.29	49.76; 5.78–11.08	99.57; 6.25–18.4	149.33; 6.55–24.87	199.05; 6.8–31.28
5	0	0.8	0	5.38; 3.74–5.98	9.33; 4.10–8.61	12.65; 4.27–10.72	15.87; 4.39–12.34	16.29; 5.12–13.82	26.61; 5.41–18.91	35.56; 5.59–22.55	43.18; 5.71–24.99
5	0	0.8	0.4	5.38; 3.75–6.25	9.33; 4.11–9.18	12.65; 4.28–11.20	15.87; 4.40–12.98	16.29; 5.15–14.92	26.61; 5.46–20.54	35.56; 5.66–24.33	43.18; 5.81–27.09
0	0.15	0.2	0	49.54; 5.01–5.04	99.1; 5.42–5.48	148.68; 5.67–5.74	198.23; 5.85–5.93	50.00; 5.67–5.68	100.00; 6.06–6.07	150.00; 6.3–6.32	199.99; 6.48–6.50
0	0.15	0.2	0.4	49.54; 5.04–5.08	99.10; 5.45–5.51	148.68; 5.69–5.79	198.23; 5.88–5.99	50.00; 5.70–5.71	100.00; 6.09–6.11	150.00; 6.33–6.35	199.99; 6.50–6.53
0	0.15	0.5	0	34.27; 4.85–5.01	66.14; 5.29–5.61	96.47; 5.58–6.10	125.47; 5.79–6.55	48.61; 5.73–5.78	96.66; 6.22–6.34	144.60; 6.54–6.72	192.25; 6.81–7.04
0	0.15	0.5	0.4	34.27; 4.89–5.14	66.14; 5.36–6.00	96.47; 5.69–6.82	125.47; 5.97–7.75	48.61; 5.79–5.89	96.66; 6.30–6.50	144.60; 6.65–7.03	192.25; 7.00–7.71
0	0.15	0.8	0	1.38; 1.05–1.05	1.63; 1.53–1.54	1.90; 1.98–1.98	2.23; 2.40–2.41	3.30; 3.79–3.81	5.33; 4.39–4.41	6.87; 4.62–4.65	8.58; 4.77–4.83
0	0.15	0.8	0.4	1.38; 1.05–1.05	1.63; 1.53–1.54	1.90; 1.98–1.98	2.23; 2.4–2.41	3.30; 3.79–3.83	5.33; 4.40–4.45	6.87; 4.62–4.68	8.58; 4.79–4.89
0	0.3	0.2	0	47.74; 5.01–5.05	95.66; 5.44–5.52	143.3; 5.70–5.80	191.15; 5.88–6.02	49.93; 5.68–5.69	99.88; 6.08–6.10	149.84; 6.34–6.36	199.78; 6.52–6.56
0	0.3	0.2	0.4	47.74; 5.04–5.10	95.66; 5.47–5.57	143.3; 5.73–5.89	191.15; 5.94–6.16	49.93; 5.72–5.73	99.88; 6.12–6.15	149.84; 6.37–6.41	199.78; 6.56–6.60
0	0.3	0.5	0	20.73; 4.57–4.67	38.78; 4.95–5.17	55.54; 5.20–5.52	71.67; 5.40–5.85	41.64; 5.66–5.73	81.10; 6.14–6.30	120.36; 6.48–6.74	158.43; 6.72–7.09
0	0.3	0.5	0.4	20.73; 4.60–4.77	38.78; 5.01–5.39	55.54; 5.28–5.85	71.67; 5.53–6.44	41.64; 5.72–5.85	81.10; 6.24–6.57	120.36; 6.67–7.33	158.43; 7.11–8.22

*Note:* The presented results are the average of 1000 repetitions.
