# Supplementary material for: A Regularized MANOVA Test for Semicontinuous High‐Dimensional Data
Source: Biom J. 2025 Apr 29;67(3):e70054. doi: 10.1002/bimj.70054 (PMC12041841; doi:10.1002/bimj.70054)
Supplement: Supplementary file 1 — Supporting information [file BIMJ-67-e70054-s001.zip › Replicability/README.pdf]

README related to the manuscript "A regularized MANOVA test for semicontinuous high-dimensional data", by Sabbioni E., Agostinelli C., Farcomeni A.

Corresponding author : Elena Sabbioni, elena.sabbioni@polito.it

Structure of the folder: The main folder `Replicability` contains the following files:

- `Results_CHEN`: folder with the results of the simulation study using the method described by Chen et al.;
- `Results_semicontinuousMANOVA`: folder with the results of the simulation study and of a simulated scenarios that mimic the real micro RNA dataset, when using the regularized MANOVA test;
- `analyzeResults_CHEN.R`: file used to merge together the results of the different Monte Carlo repetitions obtained using Chen et al. approach on the simulated data;
- `analyzeResults_semicontinuousMANOVA.R`: file used to merge together the results of the different Monte Carlo repetitions obtained using the regularized MANOVA test on the simulated data;
- `functionsSim.R`: file that contains necessary functions to run the simulation study for the regularized MANOVA test;
- `functionsSim_CHEN.R`: file that contains necessary functions to run the simulation study for the test of Chen et al.;
- `functionSim_real.R`: file that contains necessary functions to run the test on the simulated scenarios that mimic the real micro RNA dataset;
- `main_CHEN.R`: main file that performs the simulation study for the test by Chen et al.;
- `main_semicontinuousMANOVA.R`: main file that performs the simulation study for the regularized MANOVA test;
- `microRNA_sim.R`: file used to perform the regularized MANOVA test on the simulated scenarios that mimic the real micro RNA dataset;
- `microRNA_sim.RData`: it contains the sample parameters of the real micro RNA dataset;
- `semicontMANOVA_0.2.tar-gz`: semicontMANOVA package;
- `table1-2-3.R`: file used to produce Table 1, 2, 3 of the manuscript;
- `table4.R`: file used to produce Table 4 of the manuscript;
- `table5-6.R`: file used to produce Table 5 and 6 of the manuscript;

Simulation study [`"main_semicontinuousMANOVA.R"`, `"main_CHEN.R"`]: The results of the simulation study using the regularized MANOVA test can be reproduced running the file `"main_semicontinuousMANOVA.R"`, while the results of the method described by Chen et al. can be obtained running `main_CHEN.R"`. The directory is automatically set to the current working directory. The necessary packages are `"semicontMANOVA"` (available for the installation as tar.gz file

in the "Replicability" folder) and "parallel". This file load automatically also the necessary functions for the simulation ("functionSim.R" and "functionSim_CHEN.R" respectively).
The simulations were run in parallel on a cluster to reduce the time. It is possible to set the number of available cpus through the parameters ncpus and mc.cores. If there is no possibility to parallelize the code, set ncpus = 1 and mc.cores = 1.
The output of the simulation study is stored in new folders, called "Results_semicontinuousMANOVA" and "Results_CHEN" respectively. In the first folder we have two subfolders, K2, with the results obtained when comparing two groups, and K4, when we analize 4 groups. In the Results_CHEN we find just the subfolder K2, since this approach can be applied just when comparing two different groups. In each of these subfolders, we find other folders:

- H0: storing the results for the simulations under the null hypothesis;
- H1_1 − 1: storing the results for the simulations under the alternative hypothesis with $c_1 = 1$, $c_2 = 0$;
- H1_1 − 5: storing the results for the simulations under the alternative hypothesis with $c_1 = 5$, $c_2 = 0$;
- H1_2 − 0.15: storing the results for the simulations under the alternative hypothesis with $c_1 = 0$, $c_2 = 0.15$;
- H1_2 − 0.3 : storing the results for the simulations under the alternative hypothesis with $c_1 = 0$, $c_2 = 0.30$;

In each of these folders, there are different files for the different scenarios of n, p, π_{j1} and ρ that have been explored. For the regularized MANOVA results, each file contains a row for each Monte Carlo repetition, storing:

- the index of the repetition (used to set the seed);
- the time taken to run the test (5 entrances, it can be different if the code runs on a different computer);
- results of the test:
  - log-likelihood under no hypothesis $l^{\boldsymbol{\lambda}}$,
  - log-likelihood under the null hypothesis $l^{\boldsymbol{\lambda}_0}$,
  - selected value of regularization parameter under no hypothesis $\hat{\lambda}$,
  - selected value of regularization parameter under the null hypothesis $\hat{\lambda}_0$,
  - model complexity measure under no hypothesis,
  - model complexity measure under the null hypothesis,
  - Information criteria under no hypothesis $M(\hat{\boldsymbol{\lambda}}, \hat{\boldsymbol{\pi}}, \hat{\boldsymbol{\mu}}, \hat{\boldsymbol{\Sigma}})$,
  - Information criteria under the null hypothesis $M(\hat{\boldsymbol{\lambda}}_0, \hat{\boldsymbol{\pi}}_0, \hat{\boldsymbol{\mu}}_0, \hat{\boldsymbol{\Sigma}}_0)$,
  - test statistic $D^{\hat{\boldsymbol{\lambda}}, \hat{\boldsymbol{\lambda}}_0}$,
  - p-value of the permutation test,

  - final number of components p^*,
  - final number of components under H_0 (it is equal to p^*).

On the other hand, for the results by Chen et al., each file contains a row for each Monte Carlo repetition, storing:

- the index of the repetition (used to set the seed);
- p-value of the permutation test.

Tables ["table1-2-3.R", "table4.R", "table5-6.R"]: Table 1, 2, 3 show the proportion of rejections of the simulated study when $K = 2$, comparing the results of the regularized MANOVA and of the method proposed by Chen et al. To obtain it, run file "table1-2-3.R". Table 4 shows the proportion of rejections of the regularized MANOVA on the simulated study when $K = 4$. To obtain it, run file "table4.R". Table 5 and 6 show the mean number of components p^* and the mean values of $\hat{\lambda}$ and $\hat{\lambda}_0$ when applying the regularized MANOVA, with $K = 2$ and $K = 4$ respectively. To obtain it, run file "table5-6.R". All the files require package "xtable".

Real data [microRNA_sim.R]: The real data used for the two applications are confidential and can not be shared. Hence we have simulated two scenarios based on the real dataset with RNA differential expression in blastocyst cultures. The sample mean, the sample variance and covariance matrix and the probability of a missing values of the real dataset, both under the null hypothesis and under the alternative hypothesis, are contained in "microRNA_sim.RData". These scenarios are described in Section 3 of the manuscript and the test in these cases can be obtained running the file "microRNA_sim.R". This file requires the packages "semicontMANOVA" and "parallel". The results of this simulation are saved in the "Results_semicontinuousMANOVA\K2\H0_microRNA" and "Results_semicontinuousMANOVA\K2\H1_microRNA" respectively, depending if the data are generated according to the null hypothesis or not.

Session info

```
>       sessionInfo()
R version 4.1.2 (2021-11-01)
Platform: x86_64-pc-linux-gnu (64-bit)
Running under: CentOS Linux 7 (Core)

Matrix products: default
BLAS:    /usr/lib64/libblas.so.3.4.2
LAPACK: /usr/lib64/liblapack.so.3.4.2

locale:
 [1] LC_CTYPE=en_US.UTF-8       LC_NUMERIC=C
 [3] LC_TIME=en_US.UTF-8        LC_COLLATE=en_US.UTF-8
 [5] LC_MONETARY=en_US.UTF-8    LC_MESSAGES=en_US.UTF-8
 [7] LC_PAPER=en_US.UTF-8       LC_NAME=C
 [9] LC_ADDRESS=C               LC_TELEPHONE=C
[11] LC_MEASUREMENT=en_US.UTF-8 LC_IDENTIFICATION=C
```

```
attached base packages:
[1] parallel  stats     graphics  grDevices utils     datasets  methods
[8] base

other attached packages:
[1] semicontMANOVA_0.2

loaded via a namespace (and not attached):
[1] compiler_4.1.2   mvtnorm_1.1-3    matrixcalc_1.0-6
```
